# Engineering of ZnO/rGO towards NO_2_ Gas Detection: Ratio Modulated Sensing Type and Heterojunction Determined Response

**DOI:** 10.3390/nano13050917

**Published:** 2023-03-01

**Authors:** Donglin Li, Junfeng Lu, Xuanji Zhang, Dingfeng Jin, Hongxiao Jin

**Affiliations:** Zhejiang Province Key Laboratory of Magnetism, College of Materials Science and Engineering, China Jiliang University, Hangzhou 310018, China

**Keywords:** p–n junctions, ZnO, reduced graphene oxide, rGO, NO_2_ sensing

## Abstract

Nanoscale heterostructured zinc oxide/reduced graphene oxide (ZnO/rGO) materials with p–n heterojunctions exhibit excellent low temperature NO_2_ gas sensing performance, but their doping ratio modulated sensing properties remain poorly understood. Herein, ZnO nanoparticles were loaded with 0.1~4% rGO by a facile hydrothermal method and evaluated as NO_2_ gas chemiresistor. We have the following key findings. First, ZnO/rGO manifests doping ratio-dependent sensing type switching. Increasing the rGO concentration changes the type of ZnO/rGO conductivity from n-type (<0.6% rGO) to mixed n/p -type (0.6~1.4% rGO) and finally to p-type (>1.4% rGO). Second, interestingly, different sensing regions exhibit different sensing characteristics. In the n-type NO_2_ gas sensing region, all the sensors exhibit the maximum gas response at the optimum working temperature. Among them, the sensor that shows the maximum gas response exhibits a minimum optimum working temperature. In the mixed n/p-type region, the material displays abnormal reversal from n- to p-type sensing transitions as a function of the doping ratio, NO_2_ concentration and working temperature. In the p-type gas sensing region, the response decreases with increasing rGO ratio and working temperature. Third, we derive a conduction path model that shows how the sensing type switches in ZnO/rGO. We also find that p–n heterojunction ratio (*n*_p–n_/*n*_rGO_) plays a key role in the optimal response condition. The model is supported by UV-vis experimental data. The approach presented in this work can be extended to other p–n heterostructures and the insights will benefit the design of more efficient chemiresistive gas sensors.

## 1. Introduction

The emission of harmful gases (NO_x_) has a serious impact on the physical and mental health of humans. There is an urgent need for reliable methods to monitor toxic gases in the air in real time. Gas sensors have attracted a great deal of attention for applications in atmospheric monitoring, medical diagnostics, and the detection of volatile organic compounds (VOCs) [1,2,3]. Among the many gas sensors, the metal oxides semiconductor (MOS) sensors have been widely study due to its high response, low production costs and long-term stability [4].

Zinc oxide (ZnO) is an important n-type MOS material with a bandgap of 3.37 eV [5]. It is widely used in chemiresistive gas sensor [6,7] due to excellent chemical and physical properties such as high binding energy, high electron mobility, and non-toxicity to the environment [8,9,10,11,12]. It exhibits excellent gas-sensitive properties towards NO_2_ [13,14]. However, ZnO is often limited due to their high optimum working temperature (150 to 300 °C). Employing heterostructure is one of the effective ways to decrease the working temperature and enhance the sensor response. To satisfy the requirements of low working temperature with enhanced response [15], reduced graphene oxide (rGO) is often doped on ZnO to form rGO/ZnO heterostructure with p–n heterojunction. Besides its high surface area, electron mobility, chemical stability, and conductivity [16,17,18,19,20,21,22,23], rGO holds many defect sites [24,25] providing more adsorption sites for gas sensing. Sensors based on ZnO/rGO have attracted much attention due to the synergistic effect of ZnO and rGO [26,27,28,29,30,31].

In ZnO/rGO heterostructure, rGO content plays important role in gas sensing performance [32,33,34,35]. The sensing type of ZnO/rGO composites switch from n-type to p-type with increasing doping of rGO. It is divided into three working intervals: (1) With little rGO doping, the material shows n-type response. For example, Cao et al. prepared four ZnO/rGO composites and reported parts per billion (ppb) NO_2_ gas sensing with n-type response [36]. (2) With a medium rGO doping, the material exhibits mixed n- and p-type sensing properties. As shown in our previous study [37], the ZnO/rGO composite exhibits reversible switching from p- to n-type sensing which modulated by NO_2_ gas concentration and temperature. (3) With higher rGO loading, the ZnO/rGO is expected to exhibit a p-type response. The whole procedures are rarely investigated and remains challenging to uderstand. Therefore, the systematic research on the sensing performance caused by the ratio variation is essential for rational design of hybrid materials with heterojunctions exhibiting enhanced sensing property.

Herein, we systematically study rGO doping ratio-dependent sensing type switching of ZnO/rGO materials. ZnO nanoparticles were loaded with 0.1~4% rGO by a facile hydrothermal method and evaluated as NO_2_ gas chemiresistor. Increasing the rGO concentration changes the type of ZnO/rGO conductivity from n-type (<0.6% rGO) to mixed n/p-type (0.6~1.4% rGO) and finally to p-type (>1.4% rGO). Different sensing regions exhibit different sensing characteristics. In the n-type NO_2_ gas sensing region, all the sensors exhibit the maximum gas response at the optimum working temperature. In the mixed n/p-type region, the material displays abnormal reversal from n- to p-type sensing transitions as a function of the doping ratio, NO_2_ concentration and working temperature. In the p-type gas sensing region, the response decreases with increasing rGO and working temperature. Based on our observation, we derive a conduction path model that shows how the sensing type switches in ZnO/rGO with increasing of rGO doping. We also find that p–n heterojunction ratio (*n*_p–n_/*n*_rGO_) plays a key role in the optimal response condition.

## 2. Materials and Methods

### 2.1. Materials

Potassium permanganate, hydrogen peroxide, and [Zn (CH_3_COO)_2_·2H_2_O] were purchased from Lingfeng Chemical Reagent Co., Ltd., Shanghai, China. Absolute ethanol and hydrochloric acid were purchased from Aladdin Reagent Co., Ltd., Suzhou, China. The corresponding solutions were prepared using distilled water. All required replacement parts were analytically pure and used directly without any further purification.

### 2.2. Preparation of ZnO Nanoparticles and rGO

The graphene oxide used in this article was prepared by Hummers’ method [38].

1.09 g of [Zn (CH_3_COO)_2_·2H_2_O] was mixed into methanol solvent to prepare a 0.1 M solution. The solution stirred in an oil bath at 70 °C, 0.5 M sodium hydroxide solution was added dropwise to the solution until the pH = 8 and a white precipitate appeared. The precipitate was kept at 120 °C for 4 h. The resulting product was calcined at 600 °C for 2 h to obtain ZnO nanoparticles.

The 1.0 g of ZnO nanoparticles and a certain amount of graphene oxide were added to 30 mL of deionized water. The mixture was placed in an ultrasonic stirrer for 30 min, followed by stirring with a magnetic stirrer for 30 min. After the mixture was placed into a hydrothermal autoclave with 50 mL PTFE liner, it was sealed and placed at 180 °C for 9 h. After the reaction kettle was cooled to room temperature, the product was removed, washed, and then dried. These samples were labeled as ZnO-x rGO (x = 0, 0.1, 0.2, 0.4, 0.6, 0.8, 1.0, 1.2, 1.4, 1.6, 2.0, 3.0, 4.0%).

### 2.3. Material Characterization

The crystal structure and phase purity of the prepared products were studied by X-ray diffraction (XRD, Holland Panalytical, Almelo, Holland. PRO PW3040/6: X-ray source Cu Kα, working voltage 30 KV, current 25 mA). The microstructure and micromorphology of the samples were observed by field emission scanning electron microscopy (FESEM, S-4800. Hitachi Limited, Tokyo, Japan) and high-resolution transmission electron microscopy (HRTEM, JEOL-2010. JEOL, Takashima, Tokyo, Japan). The optical absorption properties of the samples were characterized by UV-Vis diffuse reflectance spectrophotometry (UV-Vis Spectrophotometer, UV-2600. Shimadzu, Osaka, Japan). The chemical composition was analyzed by X-ray photoelectron spectroscopy (XPS, Thermo Fisher Scientific, New York, NY, USA. ESCALAB 250 Xi spectrometer).

### 2.4. Gas-Sensing Measurements

First, the ZnO/rGO composite powder was mixed with an appropriate amount of ethanol and ground well with a mortar and pestle. It was evenly applied to the interdigitated electrode with a brush and then dried at 80 °C for 6 h. Finally, it was placed in the CGS-4TPS measurement system (Alitech Technology Co., Ltd., Beijing, China) for testing. The change in the resistance of the sensor was recorded by varying the concentration of the incoming gas and the ambient temperature.

## 3. Results

### 3.1. Materials Characterization

**Figure 1:**
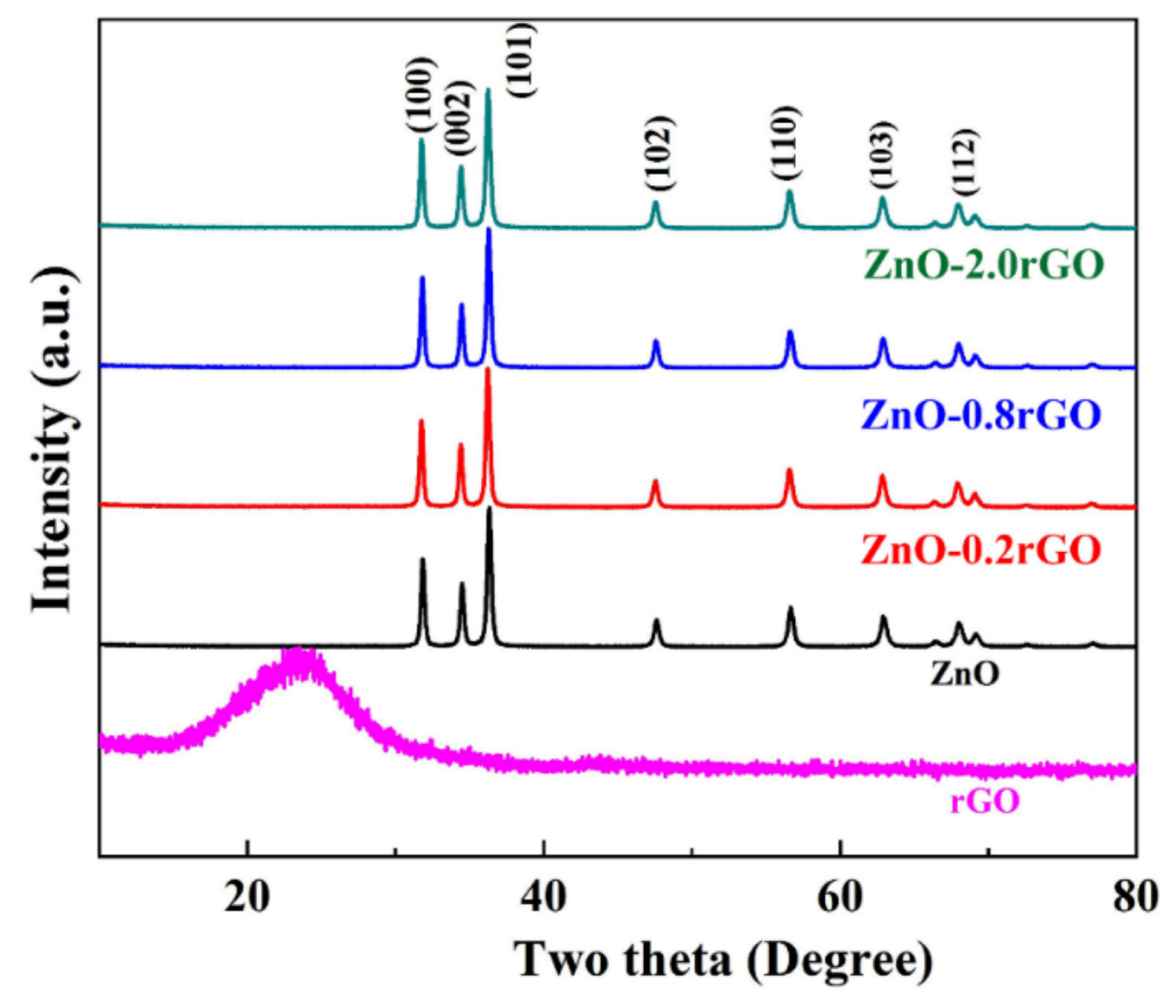
XRD patterns of rGO, ZnO, ZnO-0.2 rGO, ZnO-0.8 rGO, ZnO-2.0 rGO.

Figure 1 depicts the XRD patterns of rGO, ZnO, ZnO-0.2 rGO, ZnO-0.8 rGO, and ZnO-2.0 rGO. For rGO, there is only a broad diffraction peak (002) at 25°. The weak broad peak in rGO disappears in the ZnO/rGO due to the small content and high dispersion of rGO in ZnO/rGO. And the ZnO and ZnO-rGO nanocomposites with various rGO contents show high intensity of the diffraction peaks indicating a good crystallinity of the materials. The peaks at 31.9, 34.6, 36.5, 47.7 and 56.7° are assigned to (100), (002), (101), (102) and (110) planes of hexagonal wurtzite zinc oxide (JCPDS-36-145) [39,40]. No change in diffraction peaks of the ZnO-rGOs with that of ZnO indicates surface hybridization of rGO on ZnO because the lattice constant of ZnO remains unchanged.

**Figure 2:**
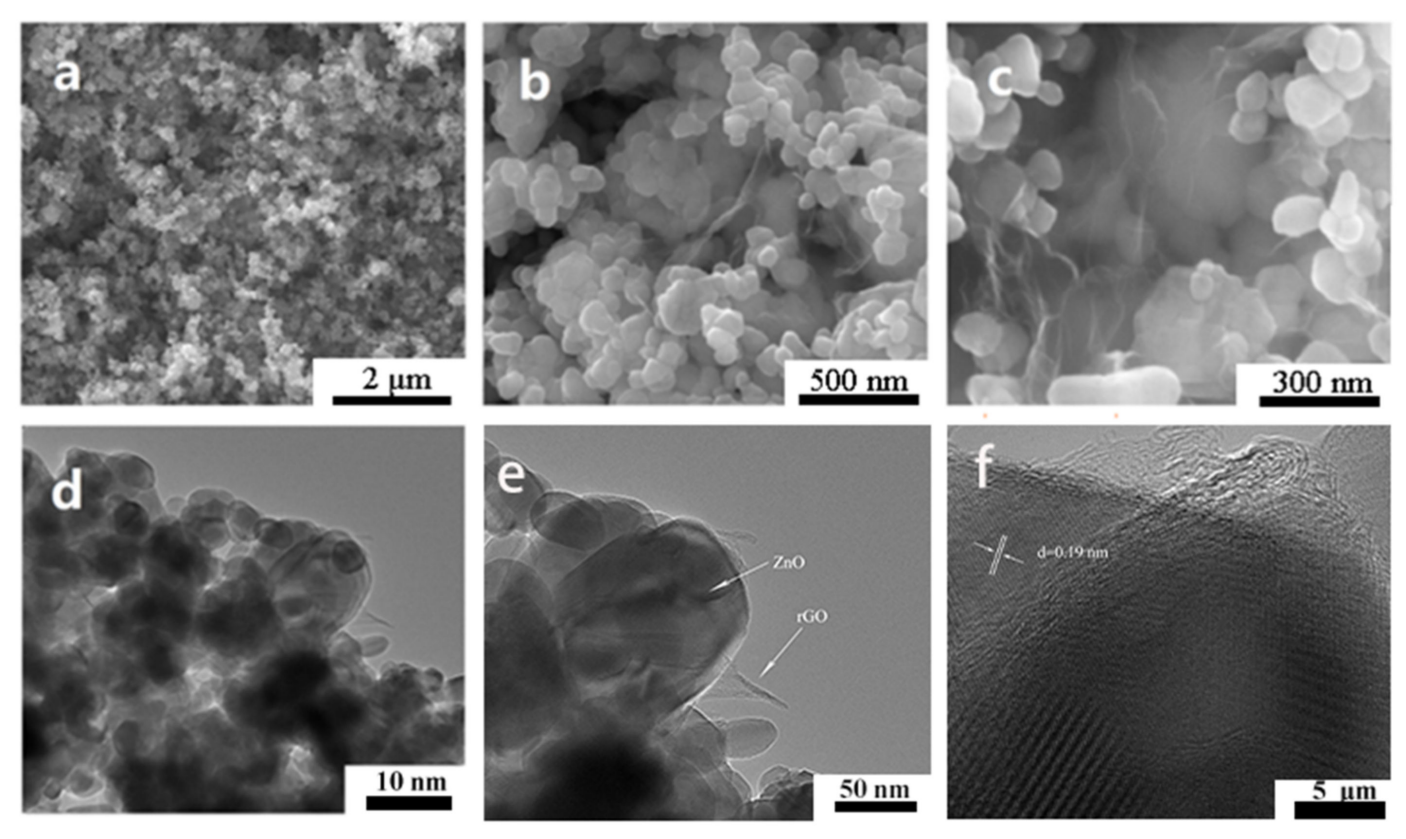
SEM images of ZnO antiparticles (**a**) and ZnO-0.2 rGO (**b**,**c**); TEM images of ZnO-0.2 rGO at low (**d**,**e**) and high (**f**) magnification.

The microstructure and morphology of ZnO and ZnO-rGO were analyzed by SEM and TEM. Figure 2 shows the typical results. The pure ZnO consists of 20–100 nm nanoparticles (Figure 2a). The sheet-like rGO is clearly observed after hybridization of ZnO with rGO (Figure 2b,c). TEM images (Figure 2d,e) show that the ZnO-0.2 rGO consists of ZnO nanoparticles covered with rGO. The higher magnification image (Figure 2f) shows a clear view of the interface between ZnO and rGO which offers the possibility of forming p–n junctions to improve the gas-sensing properties of the ZnO-rGO. The lattice fringes with a d-spacing of 0.19 corresponds to the (101) plane of wurtzite hexagonal structure ZnO.

**Figure 3:**
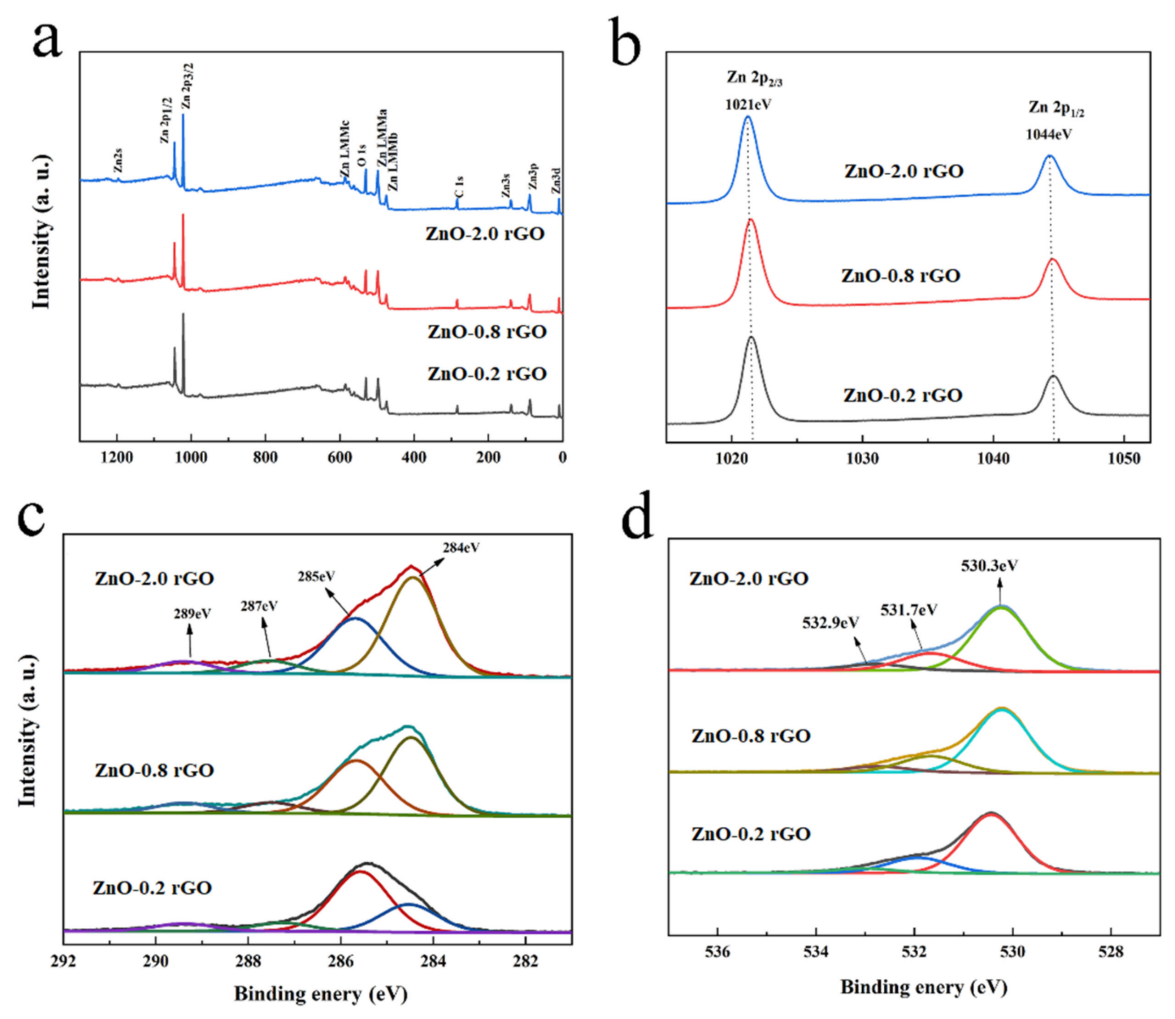
XPS spectra of ZnO-x rGO (x = 0.2, 0.8, 2.0): (**a**) survey scan spectrum; (**b**) Zn 2p spectrum; (**c**) C 1s spectrum; (**d**) O 1s spectrum.

XPS was used to confirm the chemical state of the component elements in the samples. Figure 3 shows the XPS spectra of the ZnO-x rGO (x = 0.2, 0.8, 2.0) composites. Figure 3a confirms the presence of Zn, C and O elements. The characteristic peaks of Zn 2p, Zn 2p_1/2_, and Zn 2p_3/2_ are exhibited at around 1196 eV, 1045 eV, and 1022 eV, respectively. Figure 3b displays the Zn 2p high-resolution spectrum. The peaks at approximately 1044 eV and 1021 eV are attributed to Zn 2p_1/2_ and Zn 2p3/2, respectively [41,42]. In Figure 3c, four characteristic peaks appear at 284.53 eV, 285.65 eV, 287.28 eV and 289.43 eV, corresponding to C=C, C-C, C-O and C=O in graphene, respectively. In addition, the plot of O 1s was fitted to three sections corresponding to the lattice oxygen O of ZnO in the composite with hexagonal fibrillated zincite structure O_L_ (530.3 eV), chemisorbed oxygen O_C_ (531.7 eV), and C=O (532.9 eV). Among them, the lattice oxygen O_L_ content of the composites is around 72%, while the chemisorbed oxygen O_C_ (531.7 eV) content is around 21%.

**Figure 4:**
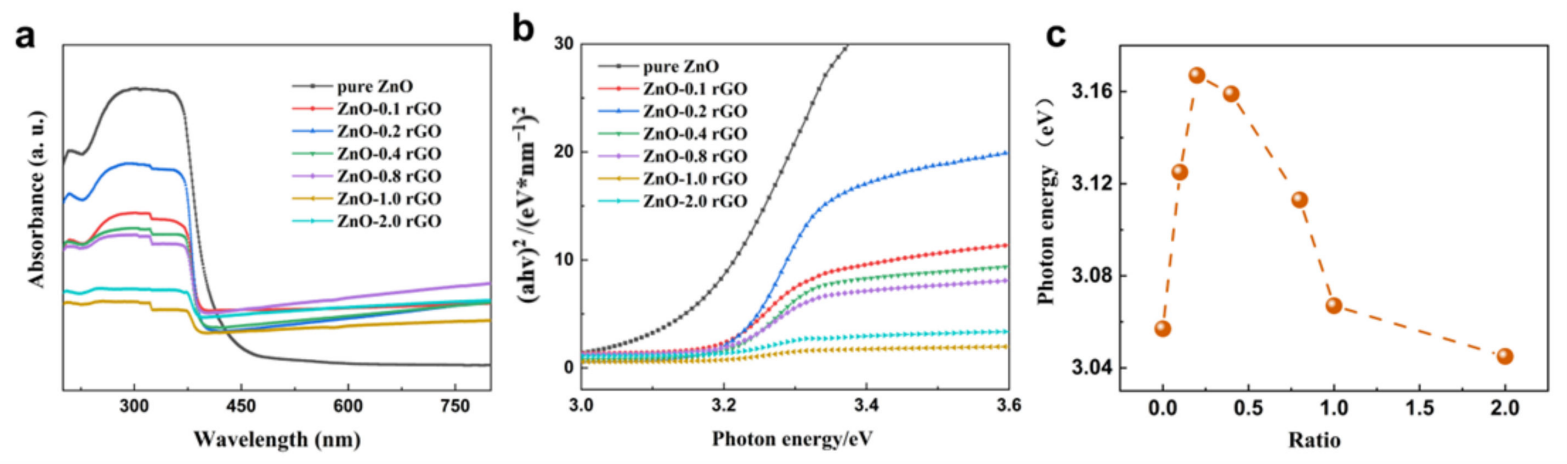
(**a**) UV-vis DRS spectra of ZnO-x rGO (x = 0, 0.1, 0.2, 0.4, 0.6, 0.8, 1.0, 2.0). (**b**) Graphical estimation of band gap; (**c**) Varation of band gap with addition of rGO.

Figure 4a shows the UV-vis DRS spectrum of the ZnO-x rGO (x = 0, 0.1, 0.2, 0.4, 0.6, 0.8, 1.0, 2.0), which shows that the presence of rGO leads to an observable shift in the spectra [43]. As shown in Figure 3b, the optical band gap of ZnO-x rGO was estimated using Equation (1).

(1) $$\mathrm{Eg}=hv-\;{\left(\frac{Ahv}{C}\right)}^{\frac{1}{2}}$$

where hv are horizontal coordinates, and ${\left(\frac{Ahv}{C}\right)}^{\frac{1}{2}}$. are vertical coordinates. The tangent line can be obtained as Eg, and *hv* can be replaced by 1240/λ, where λ is the wavelength; *A* is the degree of light absorption; and *C* is a constant. The band gap exhibits a maximum with increasing rGO doping (Figure 3c). And ZnO-0.2 rGO has a maximum optical band gap. The change in the band gap can be attributed to the interaction of Zn-O-C with ZnO-rGO. Similar results are also shown by other researchers. Puneetha J et al. [44] found that ZnO/GO nanohybrid materials have a small red shift (6 nm) compared with the pure ZnO. They ascribe those to the chemical binding (Zn-O-C) between ZnO and GO. Rahimi et al. [45] find that the Eg value decline from 3.2 eV (ZnO) to 2.8 eV for ZnO nanorod/graphene quantum dot composites respectively. They ascribe those to the chemical bonds formed such as Zn-O-C or Zn-C bonds in the composites. The larger percent of p–n junctions in the material, the wider optical band gap of is expected. In our observation, it is interesting to find that sensor based on ZnO-0.2 rGO with a maximum band gap shows the best sensitivity toward NO_2_ sensing in the n-type sensing region (Section 3.2).

### 3.2. Gas Sensing Property

**Figure 5:**
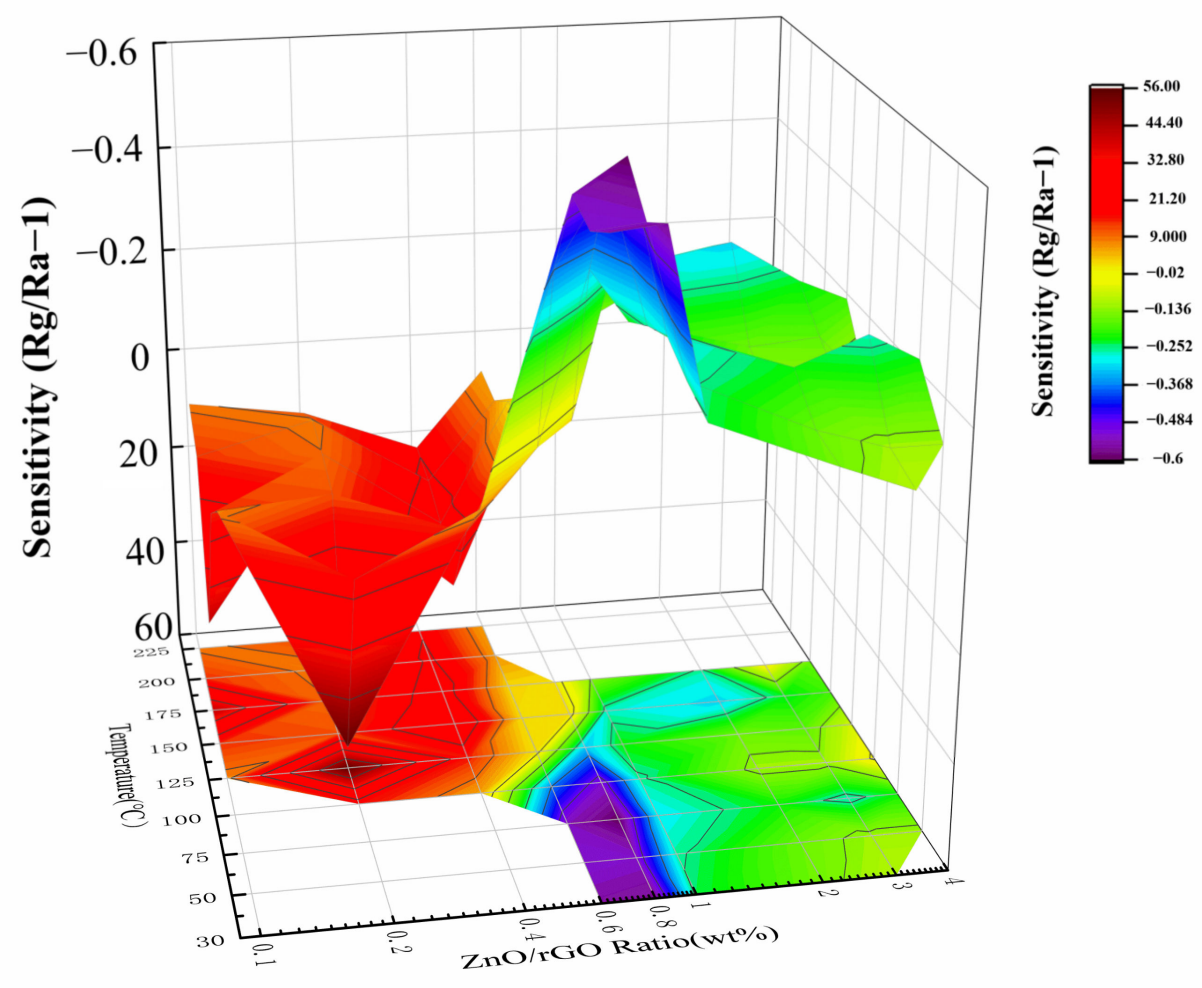
Variation of the sensitivity of the sensors base on the ZnO-x rGO with temperature and addition of rGO toward 4 ppm NO_2_.

**Figure 6:**
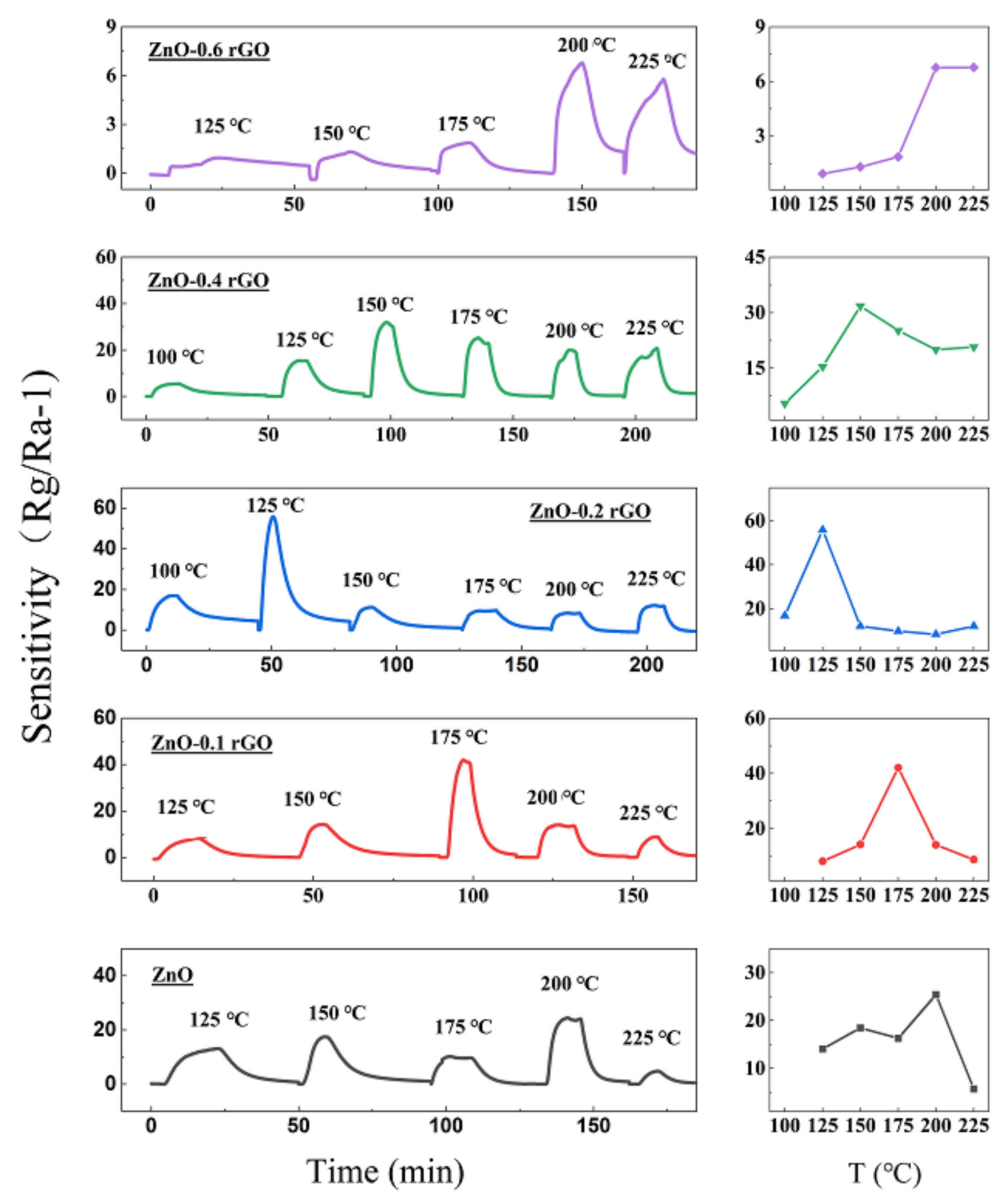
Response curves of the ZnO-x rGO (x = 0, 0.1, 0.2, 0.4, 0.6) with temperature (100–225 °C) at a concentration of 4 ppm NO_2_ on the left.

**Figure 7:**
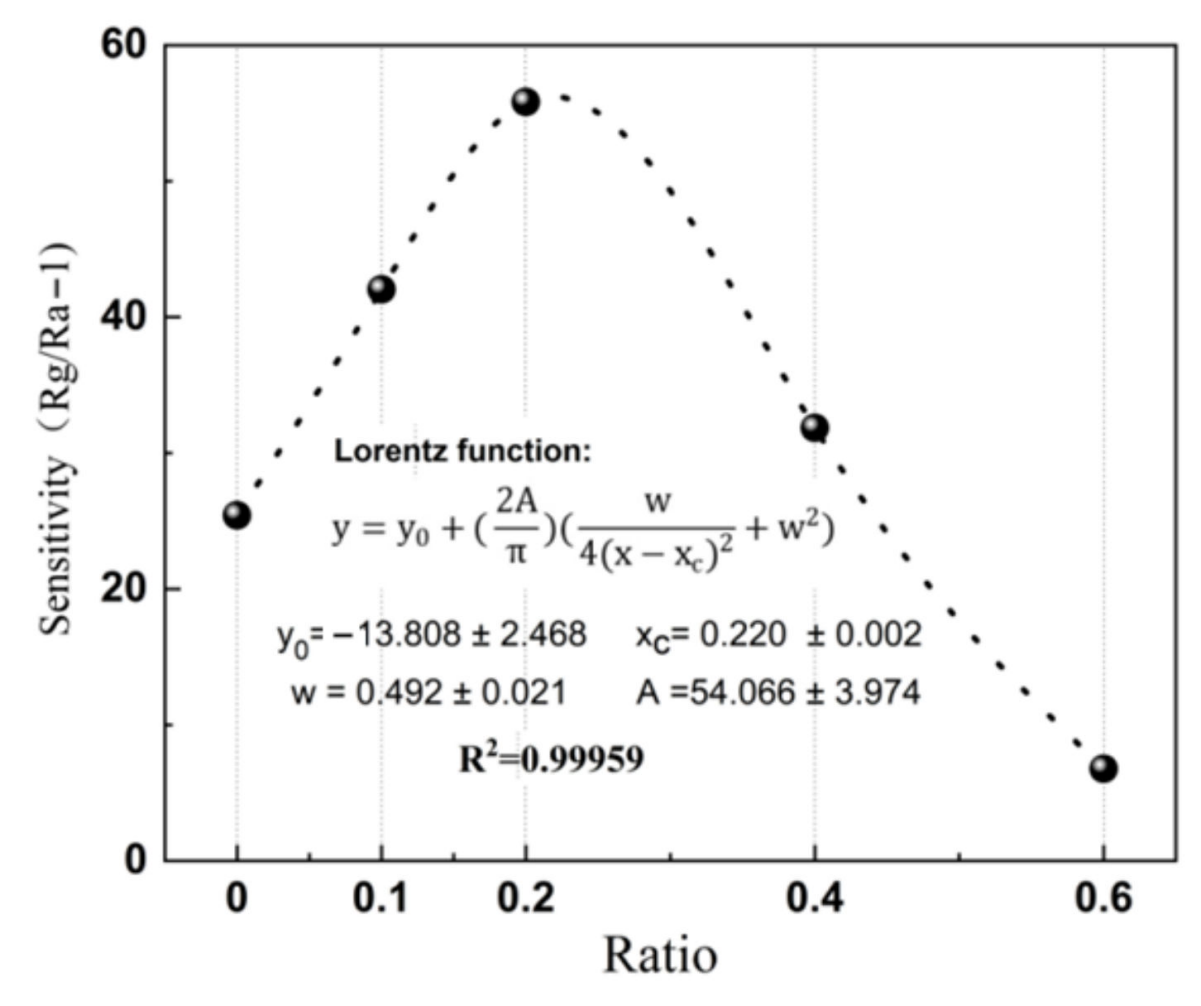
Correlation between the response of the ZnO-x rGO (x = 0, 0.1, 0.2, 0.4, 0.6) sensor and ratio.

The ZnO-x rGO samples (x = 0, 0.1, 0.2, 0.4, 0.6, 0.8, 1.0, 1.2, 1.4, 1.6, 2.0, 3.0, 4.0) obtained by the hydrothermal method were used to prepare gas sensors. The sensing properties of the composites were tested at various temperatures at different NO_2_ concentrations. The sensor sensitivity was calculated according to Equation (2).

(2) $$\mathrm{Sensitivity}=\frac{Rg}{Ra}-1$$

where *Rg* and *Ra* are the resistances of the sensing elements in the presence of NO_2_ gas and air, respectively. The sensitivity is positive (negative) which indicates that the material is an n-type (p-type) semiconductor. Figure 5 depicts the sensitivity of the samples toward 4 ppm NO_2_. The composites behave as n-type semiconductors, when the doping concentration is low (x = 0, 0.1, 0.2, 0.4, 0.6) as shown in the red area. However, as the doping concentration increases (x = 0.6, 0.8, 1.0, 1.2, 1.4) as shown in the yellow area, the sensors begin to change from p- to n- type. Finally, when the doping concentration is high (x = 1.6, 2.0, 3.0, 4.0, 5.0) as shown in the blue area, sensors exhibit p-type responses.

n-type sensing: Sensors based on the ZnO-x rGO (x = 0, 0.1, 0.2, 0.4, 0.6) show n-type conductivity (Figure 6). The resistance of the sensors increases when they come into contact with NO_2_. Small amount of rGO addition to form p–n junctions do not change the sensing type. The results showed that all sensors have an optimal temperature with maximum sensitivity. This is a consequence of competition between gas adsorption and desorption [46]. Among them, the ZnO-0.2 rGO has the highest sensitivity of 56 at 4 ppm NO_2_ with a low optimal working temperature of 125 °C. And the maximum response of the sensor to different ratio has a good nonlinear correlation, which conforms to the *Lorentz* function (Figure 7). The correlation coefficient is R^2^ = 0.99959 in the range of 0~0.6%. It means that the sensitivity is related to the charge carriers inside the material [47,48,49]. This is related to the percentage of p–n junctions in the complex as shown in UV-vis spectrum (Figure 4).

**Figure 8:**
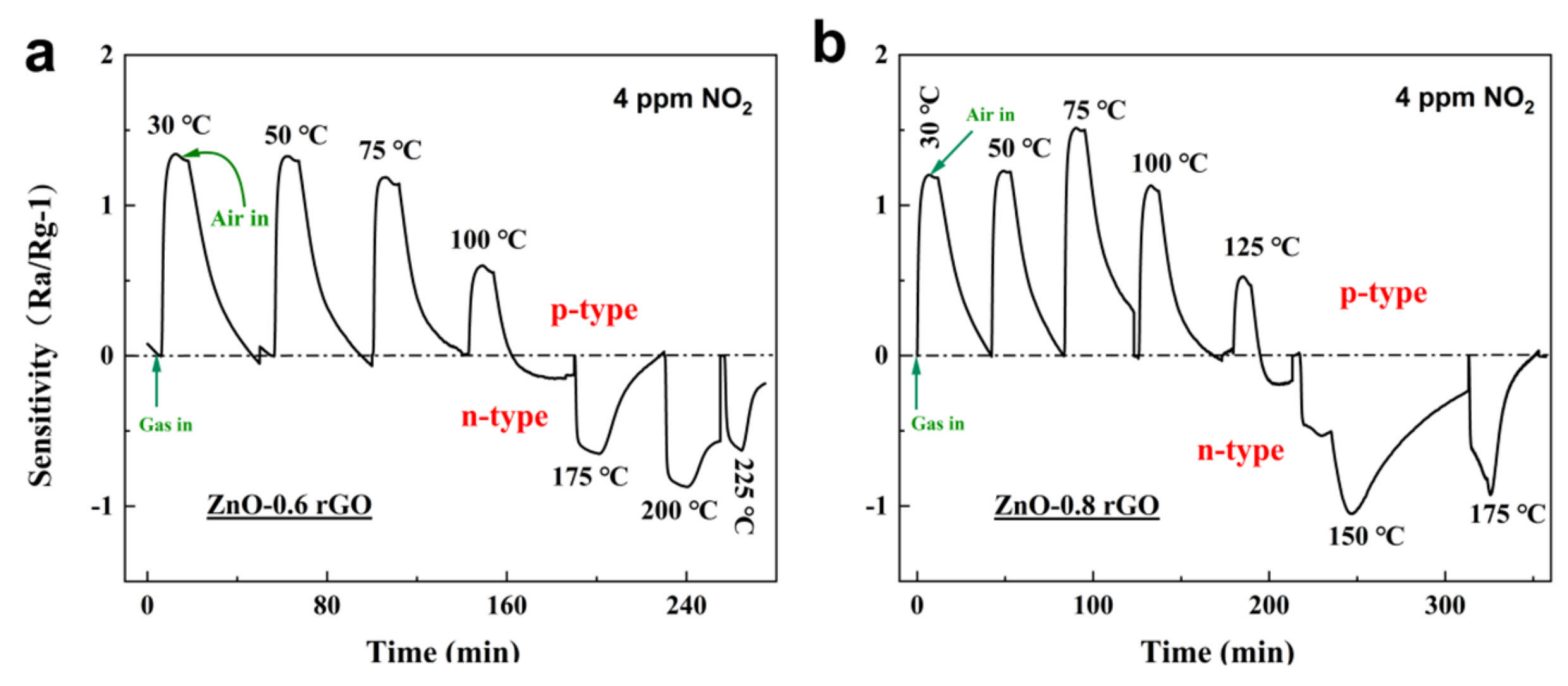
Dynamic sensing transients of (**a**) ZnO-0.6% rGO and (**b**) ZnO-0.8% rGO at 4 ppm to 30−225 °C.

**Figure 9:**
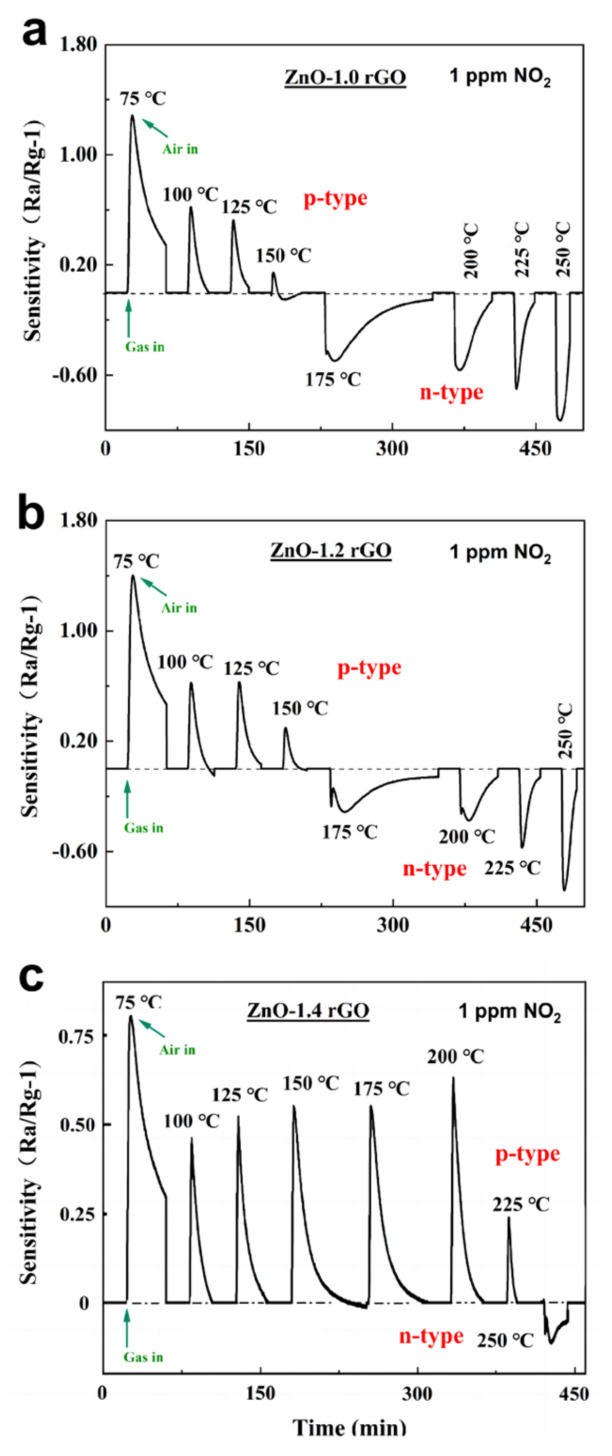
Dynamic sensing transients of (**a**) ZnO-1.0% rGO, (**b**) ZnO-1.2% rGO and (**c**) ZnO-1.4% rGO at 1 ppm to 30–225 °C.

**Figure 10:**
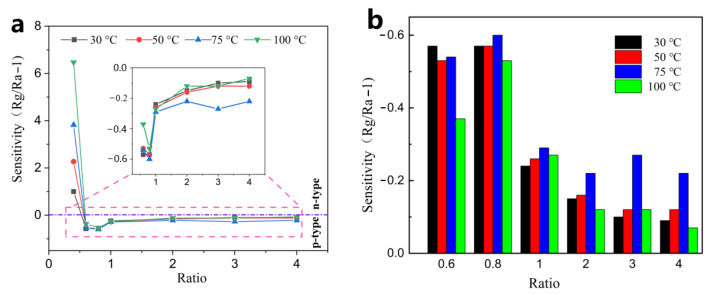
(**a**) response of the ZnO/x% rGO (x = 0.4, 0.6, 0.8, 1.0, 2.0, 3.0, 4.0) to 4 ppm at 30–100 °C; (**b**) Response curves of the ZnO/x% rGO (x = 0.6, 0.8, 1.0, 2.0, 3.0, 4.0) in the subsidiary vignette.

Switching from n- to p-Type: ZnO and rGO are n-type and p-type semiconductors, respectively. When a small amount of rGO is introduced into ZnO, the material remains an n-type semiconductor because it acts as a dopant. However, when the proportion of rGO continues to increase and reaches a threshold, the material transforms into a p-type semiconductor when rGO becomes the dominant factor. A p–n mixing region appears before the composite completely transitions to a p-type semiconductor. To find this sensing type region, sensors based on ZnO-x rGO (x = 0.6, 0.8, 1.0, 1.2, 1.4, 1.6) were tested at different operating temperatures from 50 to 250 °C at 4 ppm (Figure 8) or 1 ppm (Figure 9) NO_2_, sensor sensitivity was calculated according to Sensitivity = $\frac{Ra}{Rg}$ −1. These samples undergo p–n conversion at different test concentrations due to different doping ratios of rGO. ZnO-0.6 rGO and ZnO-0.8 rGO show the transition from p-type to n-type in 4 ppm NO_2_ gas (Figure 8). ZnO-1.0 rGO, ZnO-1.2 rGO and ZnO-1.4 rGO (Figure 9) show the p–n transition in 1 ppm NO_2_. The n- to p-type sensing transitions as a function of the doping ratio, NO_2_ concentration and working temperature can be explained by mechanisms based on charge transfer and surface reactions, as explained in our previous article [37].

p-type sensing: As the amount of rGO continues to increase and reaches a threshold, rGO becomes the dominant factor and therefore the material transforms into a p-type sensing. When the doping ratio is increased to 0.6%, the composite starts to exhibit a p-type response in the low-temperature region (30–100 °C) at 4 ppm (Figure 10). As shown in the Figure 10a, the ZnO-0.4 rGO complex is n-type response, while ZnO-0.6 rGO exhibits a p-type response. As shown in Figure 10b, ZnO-0.8 rGO exhibit the maximum value of the p-type response in the p–n transition region. The sensitivity decreases with increasing rGO doping ratio in the p-type region. Compared with n-type complexes, the optimal operating temperature of p-type materials is reduced to 75 °C.

**Figure 11:**
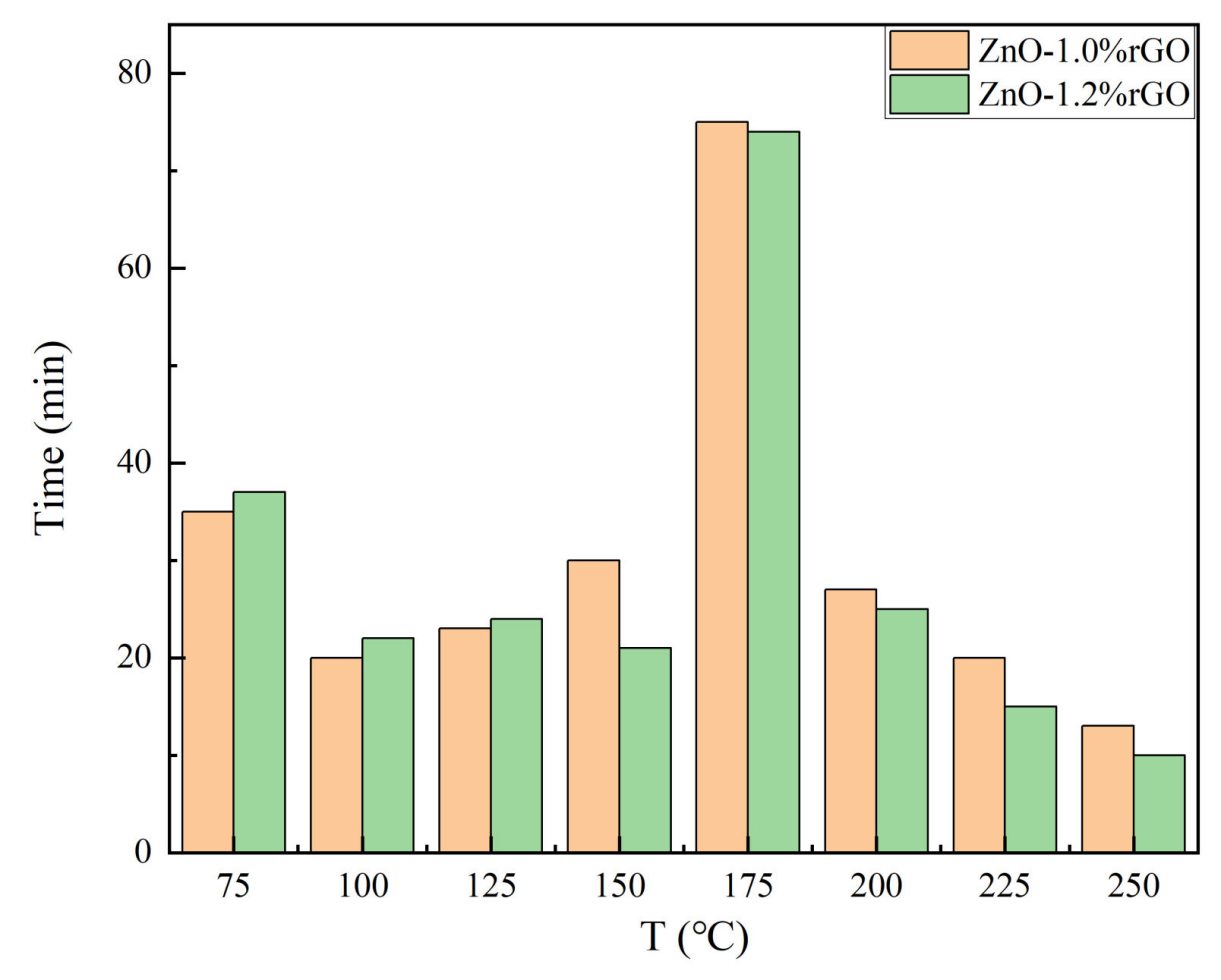
Temperature dependence of recovery time of ZnO-1.0% rGO and ZnO-1.2% rGO at 1 ppm NO_2_ gas concentration.

Recovery time: As shown in Figure 9, the recovery time of the sensing materials show a max with the increasing of temperature. In order to make a clear observation, we made a diagram (recover time VS temperature) for the two samples (ZnO-1% rGO and ZnO-2% rGO) with good response. As shown in the Figure 11, the recovery time increased first and then decreased with the increase of temperature. The material first shows p-type response and then starts to turn to n-type response. At 175 degrees, both n- and p-type sensing occur in the materials. There is competition between p-type response and n-type response, which might make the recovery time longer. At the second stage, with the further increase of the temperature, the materials display n-type sensing. Since the desorption of the gas molecular is faster at higher temperature, the recovery time decrease with the increasing of the temperature at this stage.

**Figure 12:**
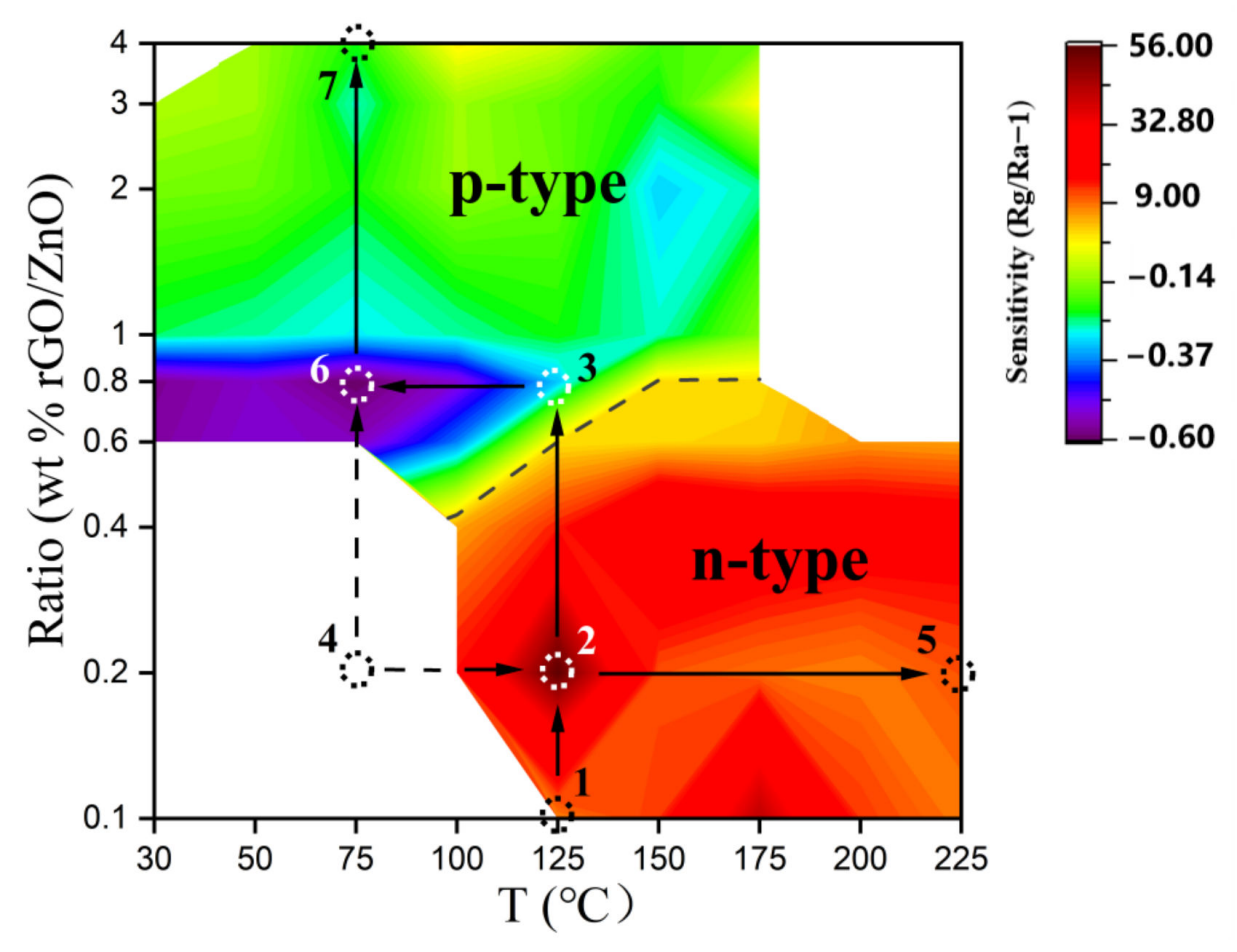
Response of sensors based on the ZnO-x rGO (x = 0.1, 0.2, 0.4, 0.6, 0.8, 1.0, 2.0, 3.0, 4.0) to 4 ppm NO_2_ gas at different temperature (30–225 °C).

Overview: In the n-type region we find the maximum sensitivity, while in the p-type region we observe that the sensitivity decreases continuously as the doping ratio of rGO increases and maximum sensitivity appears in the p–n transition region. To observe the changes in sensitivity occurring for different ratios of ZnO-x rGO (x = 0.1, 0.2, 0.4, 0.6, 0.8, 1.0, 2.0, 3.0, 4.0), we plotted the sensitivity plot with respect to temperature and doping ratio. As shown in Figure 12, it is clear from the plot that the maximum sensitivity of the n-type sensing materials is ZnO-0.2 rGO at 125 °C, while the maximum sensitivity of the p-type sensing materials is ZnO-0.8 rGO at 75 °C. It shows that n-type sensing materials exhibit optimum sensitivity at high temperature (the red area), the best working temperature of p-type material is low temperature environment (the blue area). It (Line 4-2-5) indicates a change in sensitivity with increasing temperature, which is related to the change in internal carrier concentration. The change of temperature will affect the change of electron concentration inside the material and the adsorption and desorption equilibrium on the surface [46]. It (line 1-2-3) indicates a change in sensitivity with increasing ratio. It is the result of the joint action of p–n junction and rGO. ZnO-0.2 rGO has the maximum sensitivity at 125 °C in the n-type region because the p–n junction has the greatest effect at this time and the smallest concentration of electron carriers. It (lines 4-6-7) also shows that the sensitivity decreases with increasing rGO ratio. It (line 3-6) indicates a change in sensitivity with decreasing temperature. RGO plays a major role in the whole blue region, while it plays an auxiliary role in p–n. When the temperature is 75 °C, the hole carrier of ZnO-0.8 rGO is relatively small, and the role of p–n junction is greater than that of other conditions, so it has the maximum sensitivity.

## 4. Discussion

**Scheme 1:**
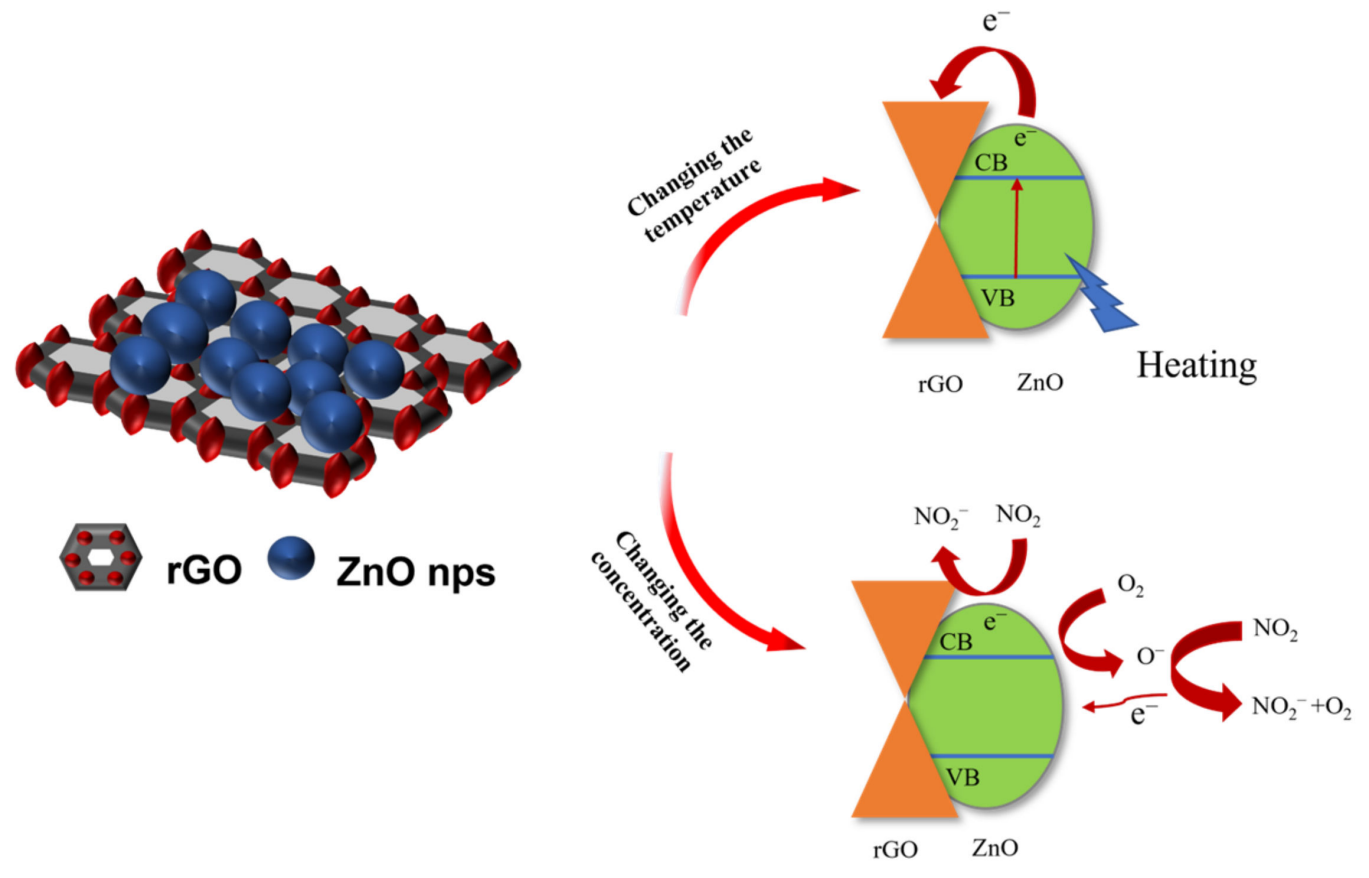
Mechanism diagram of temperature and NO_2_ gas affecting material sensitivity.

Carrier transition mechanism and surface chemical reaction are used to explain the mechanism of material sensitivity change [37]. As shown in Scheme 1, upon rGO loading, the electrons of ZnO conduction band flow to rGO and form p–n junction on the contact surface. When working temperature changes, the carrier concentration in the material and the adsorption and desorption balance on the surface of the material will change accordingly, resulting in changes in sensitivity.

Under air condition, oxygen adsorbs on the surface of the material and captures electrons from the valence band. Reaction (1) is the reaction of oxygen on the material surface. There are two different states of adsorbed oxygen: O_2_^−^ (below 100 °C) and O^−^ (100~200 °C). When NO_2_ gas molecules are adsorbed on the material surface, they are chemically adsorbed as NO_2_^−^ (reaction (2)). At the same time, some NO_2_ will react with O^−^ or O_2_^−^ (reaction (3)), leading to the change of carrier in the materials and the resistance of the materials.

$$O_{2}\left(g\right)+2e^{-}\to 2O_{\left(\mathrm{ads}.\right)}{}^{-}$$

$${\mathrm{NO}}_{2}\left(g\right)+e^{-}\to {\mathrm{NO}}_{2}{{}_{\left(\mathrm{ads}.\right)}}^{-}$$

$${\mathrm{NO}}_{2}\left(g\right)+2O_{\left(\mathrm{ads}.\right)}{}^{-}\to {\mathrm{NO}}_{2}{{}_{\left(\mathrm{ads}.\right)}}^{-}+O_{2}\left(g\right)+e^{-}$$

**Scheme 2:**
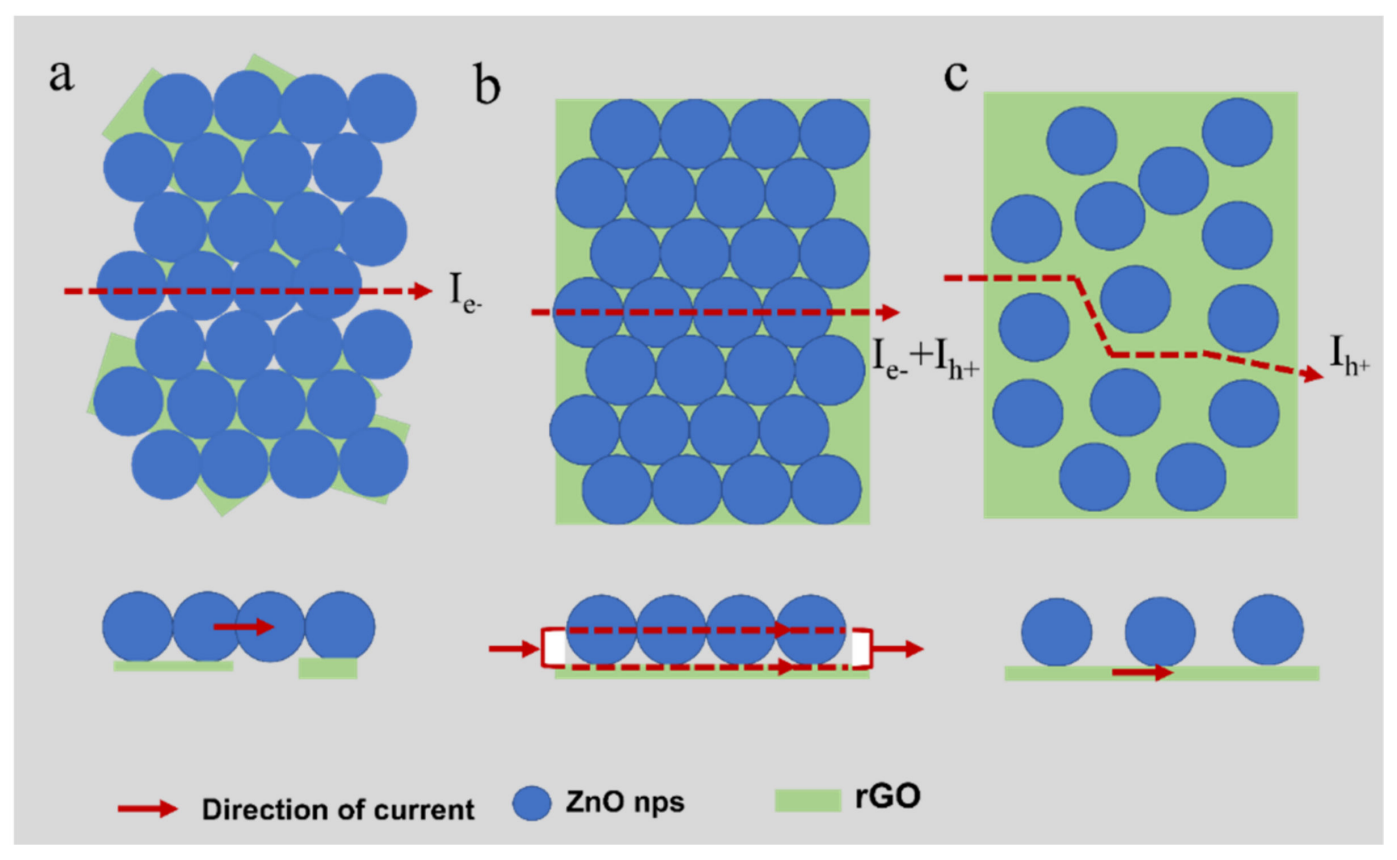
Three conductive paths: (**a**) Conducting through ZnO; (**b**) Conducting through ZnO and rGO; (**c**) Conducting through rGO.

In order to further understanding the doping ratio modulated sensing properties, the conduction path model is proposed. As shown in Scheme 2, with low and moderate graphene loading, graphene sheets are not interconnected (Scheme 2a), and the conductivity is caused by electrons (I_e−_). Graphene sheets begin to interconnect with increasing graphene doping (Scheme 2b), and the conduction path is mainly through ZnO nanoparticles and rGO (I_e−_ + I_h+_). As the rGO content further increases (Scheme 2c), graphene becomes a major part of the conductivity (I_h+_).

The difference in the conductivity path indicates a change in the sensing type in the composite. However, the key factors that affect the sensitivity of the composite are the p–n junction and rGO. The formation of the p–n junction led to extensive expansion of the depletion region when NO_2_ is adsorbed on the surface [50]. The sensor resistance increases significantly due to the deep extension of depletion regions compared to pure ZnO. The p–n junction contributes to the sensitivity of the composite. But rGO give poor response due to its weak Van der Waals interactions with NO_2_ gases [46]. Therefore, the joint effect of the p–n junction and rGO is proposed. And it is expressed as *ψ* = *n*_p–n_/*n*_rGO_, where *n*_p–n_ is the content of the p–n junction in the whole composite and *n*_rGO_ is the content of rGO in the whole composite.

**Scheme 3:**
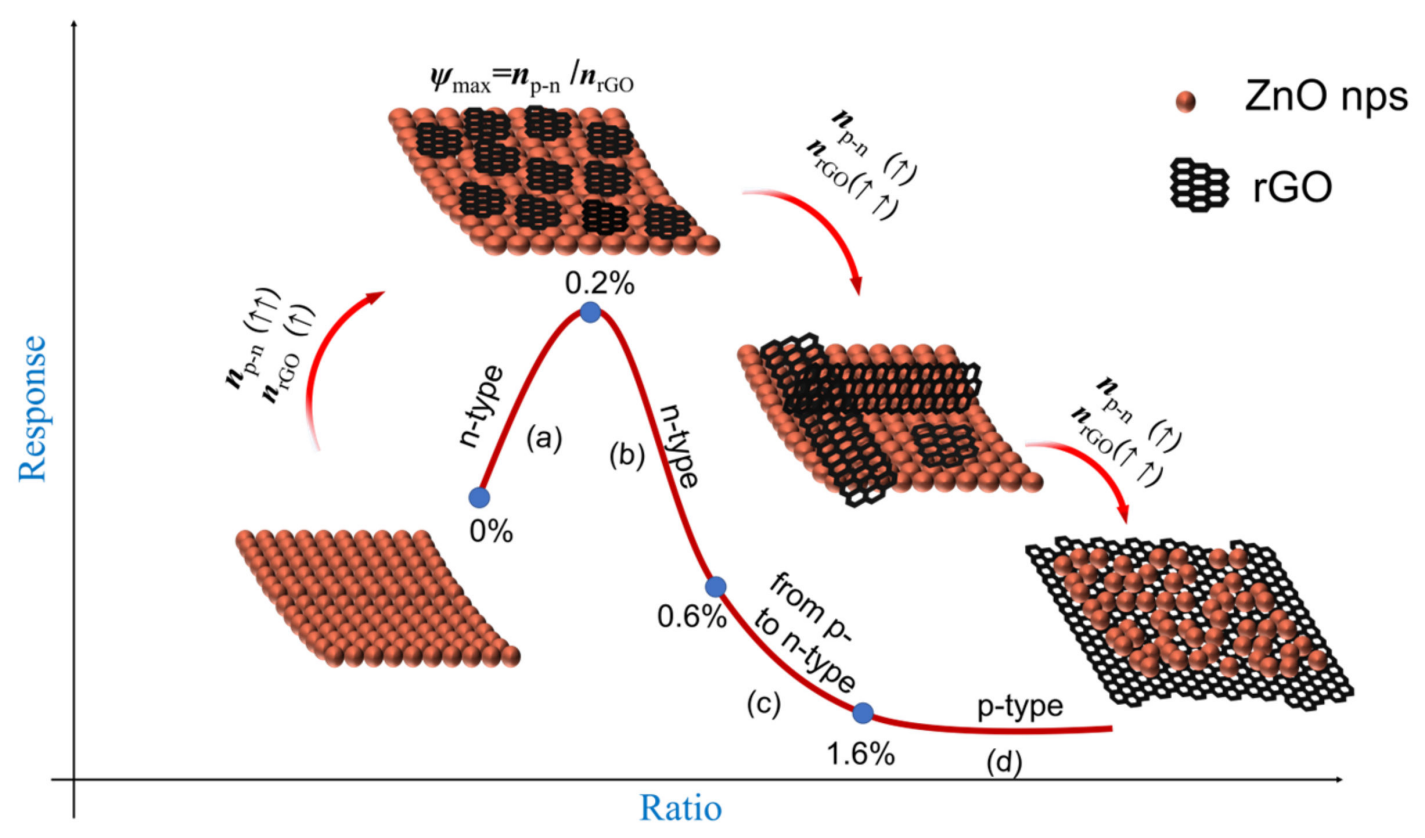
Variation of the response with the rGO doping ratio in consideration of *n*_p–n_/*n*_rGO_: (**a**) the region of the n-type response increasing with rGO loading; (**b**) the region of the n-type response decreasing with rGO loading; (**c**) the region of switching from n- to p-Type; (**d**) the region of the p-type response. *n*_p–n_: The amount of p–n junction; *n*_rGO_: the amount of rGO; (↑↑) increases faster than (↑).

Scheme 3 shows the increasing trend of *n*_p–n_ and *n*_rGO_ with increasing rGO content. In the first part (Scheme 3a), as the rGO content increases, *n*_p–n_ increases faster than *n*_rGO_. The p–n junction plays a major role in the material response when the graphene loading level is low. There can be a large number of p–n junctions at this low-weight graphene content due to the very low graphene density. *ψ* will rise (*n*_p–n_ >> *n*_rGO_). After the introduction of NO_2_, the sensor resistance increased due to the extension of the depletion regions. The sensitivity of the composite increases with increasing *ψ* when the graphene loading level is 0~0.2% (Scheme 3a). The response is optimal in the case of prepared graphene-zinc oxide composites with a graphene loading of approximately 0.2%.

The second part is that *n*_rGO_ increases faster than *n*_p–n_ as the rGO content continues to increase. The role of rGO in composites is becoming increasingly significant. The sample will undergo two processes: a. Composites will remain an n-type semiconductor (Scheme 3b); b. The semiconductor type of the composite changes from n-type to p-type with temperature or gas concentration (Scheme 3c). At higher graphene concentrations (0.4~1.4%), the dispersed conducting graphene sheets start to partially connect, resulting in a shorter resistance path through the graphene sheets and a larger dropping in resistance in air, as shown in Scheme 2b. When exposed to NO_2_, the p–n junction still plays a key role, but its content is low relative to graphene, and the conductive pathway is partly through graphene, the sensors resistance will be increased less. The sensitivity starts to decrease (Scheme 3b). At the same time, when the external conditions change, the sensing type of the material will also be transformed (Scheme 3c). When the material is a p-type semiconductor (1.6 ~4.0%), graphene becomes the only conductive path in Scheme 2c. The content of the p–n junction is much smaller than that of rGO (*n*_p–n_ << *n*_rGO_). *ψ* decreases with increasing doping of rGO, and the sensitivity of the sample also decreases. The sensitivity decreases with decreasing *ψ* at higher graphene concentrations (0.4~4.0%) (Scheme 3b–d).

As shown Scheme 3, the trend of sensitivity is to increase and then decrease. The model shows that the trend of *ψ* = *n*_p–n_/*n*_rGO_ also increases and then decreases. Meanwhile, UV-vis DRS can directly detect the trend of n in line with the trend of sensitivity, which further validates that *ψ* is a key factor affecting the sensitivity. Based on the proposed influencing factors (*ψ* = *n*_p–n_/*n*_rGO_), the same holds true for SnO_2_ /rGO. In the study by Li G D et al. [51]. The 0.2 wt% rGO-SnO_2_ nanocomposites show a higher response to 3 ppm NO_2_ at 125 °C than pure rGO, 0.1 wt% rGO-SnO_2_, 0.4 wt% rGO-SnO_2_, 0.6 wt% rGO-SnO_2_ and pure SnO_2_. The rGO-SnO_2_ complex exhibits an n-type response, and the response increases and then decreases with increasing graphene content. This result is consistent with our results. The rGO-SnO_2_ complex exhibits a p-type response. Zhu X Y et al. [52]. prepared four different ratios of rGO-SnO_2_ (rGO, rGO—1 mg/mL SnO_2_, rGO—2 mg/mL SnO_2_, rGO—2 mg/mL SnO_2_). The experimental results revealed an increased sensing response with increasing SnO_2_ concentration from 0 to 10 mg/mL. which means that the sensing response decreases as *n*_p–n_/*n*_rGO_ decreases. Similar response enhancement can be achieved if other materials can form high-density or heterogeneous junctions, and the best ratio of sensitivity is determined by the *ψ* value.

## 5. Conclusions

In summary, nanomaterial ZnO/rGO composites with different rGO loading ratio were prepared by a facile hydrothermal method. Gas sensor based on those materials were fabricated for NO_2_ detection. A series of comparison experiments confirmed that the sensing type (n-type, p-type or mix n- and p-type) was modulated by rGO ratio. Based on the observation, a conduction path model was proposed to show how the sensing type switches in the materials. And the p–n heterojunction ratio (*n*_p–n_/*n*_rGO_) of the material plays a key role in the optimal response condition. The approach presented in this work can be extended to other heterostructures and the insights will benefit the rational design of more efficient chemiresistive gas sensors.

## Data Availability

The data presented in this study are available on request from the corresponding author.
